# Is Strongyloidiasis Endemic in Spain?

**DOI:** 10.1371/journal.pntd.0003482

**Published:** 2015-02-05

**Authors:** Angela Martinez-Perez, Rogelio Lopez-Velez

**Affiliations:** National Referral Centre for Tropical Diseases, Infectious Diseases Department, Ramon y Cajal Hospital, IRYCIS, Madrid, Spain; Hitit University, TURKEY

*Strongyloides stercoralis* is a widely distributed nematode that causes human infection nearly everywhere on the globe except the poles. Although the real figures are controversial, its prevalence has been said to range from 30 to 100 million infected people [1, 2].

One of the major challenges in assessing *S. stercoralis* prevalence is the lack of well-conducted studies in suspected endemic areas. As diagnosis techniques used to detect worm infestations are not sensitive enough to detect the larvae of *S. stercoralis*, other specific techniques are required. Those techniques, such as Harada-Mori filter paper strip, Baerman concentration technique, or Koga agar plate culture, are both time and resource consuming, and newer enzyme-linked immunosorbent assay (ELISA)–based serological tests are only available in some settings, thus limiting the diagnosis in endemic areas [3].

*S. stercoralis* has a long life span and a fascinating life cycle. Rhabditiform larvae are passed in the stools of infected subjects. These develop in wet, moist soil into third-stage filariform larvae, which are infective to new hosts and can penetrate intact skin. Direct skin contact with contaminated soil is therefore a risk factor for acquisition of the infection, especially where sanitary collection systems are insufficient. Furthermore, female worms can reproduce parthenogenetically. Hence, the same human host can undergo autoinfection cycles, which may lead to prolonged infections for decades [1, 2].

Data from the last decade from Spain have shown that autochthonous strongyloidiasis is rare and restricted to one specific area [4–6]. Nevertheless, Spain seems to be considered an endemic country for strongyloidiasis, as shown in two recent reviews on the global epidemiology of this disease that were both published within one year in *PLOS Neglected Tropical Diseases* [2, 7]. Schär et al. carried out a Bayesian meta-analysis in order to obtain an estimated prevalence rate for each country with available information. They also compared risk factors among different groups at risk [2]. Puthiyakunnon et al. reviewed cases reported worldwide in an attempt to delineate a global prevalence and also provided an updated discussion on diagnostic methods and management [7].

Schär et al. found five articles describing the prevalence of *S. stercoralis* in Spain since 1989; within these articles, three studies focused on immigrants and refugees, and the other two studies were actually performed in recently arrived populations and therefore might reflect the rates from their countries of origin. Nevertheless, in an epidemiological and preventive perspective, these data are important for the country where the population is currently residing, even if they do not reflect local transmission of the infection. The other two studies were performed in a Spanish population and carried out by Roman-Sanchez et al. in a hospital-based study published in 2001 and a community-based study published in 2003 [8, 9]. When adjusted for the sensitivity of the diagnostic tool used (Koga agar plate for larval culture), an estimated model-based prevalence of 1.9% and 14.8%, respectively, was obtained, and it revealed a prevalence of ≤10% for hospital-based studies and 11%–20% for community-based ones. It is noteworthy that the authors specify the location and population type for the community-based study but fail to do so for the hospital-based one [2].

On the one hand, Puthiyakunnon et al. state the following: “Globally, prevalence rates of strongyloidiasis are as high as 50% in certain areas where moist soil and improper disposal of human waste coexist, especially in West Africa, the Caribbean, Southeast Asia, tropical regions of Brazil, Cambodia and temperate regions of Spain.” Although this statement stands for global prevalence rates and not for the individual countries listed in the second part of it, we would like to highlight that such a high prevalence has never been described anywhere in Spain so far. On the other hand, based on references 123 and 124 in their Table 1, the authors have assigned a 10%–20% prevalence rate of *S. stercoralis* infection for Spain in their Fig. 1. The legend in this figure indicates it was constructed considering “the highest percentage prevalence of reported case studies and screening among populations” [7]. Our viewpoint is that this can lead to misinterpretation of real figures.

We want to stress that both reviews have based their estimates of *S. stercoralis* prevalence in Spain by focusing on the same two studies [8, 9]. These two studies were published by the same researchers within two years and were conducted in the same geographical area of Spain, known to be endemic for *S. stercoralis*. This used to be a rice-field agricultural region, the region of La Safor; it is located in the province of Valencia within the Valencian autonomous community and contains up to 31 municipalities, Gandía and Oliva being the most populated ones with around 80,000 and 28,000 inhabitants each (Fig. 1).

**Fig 1:**
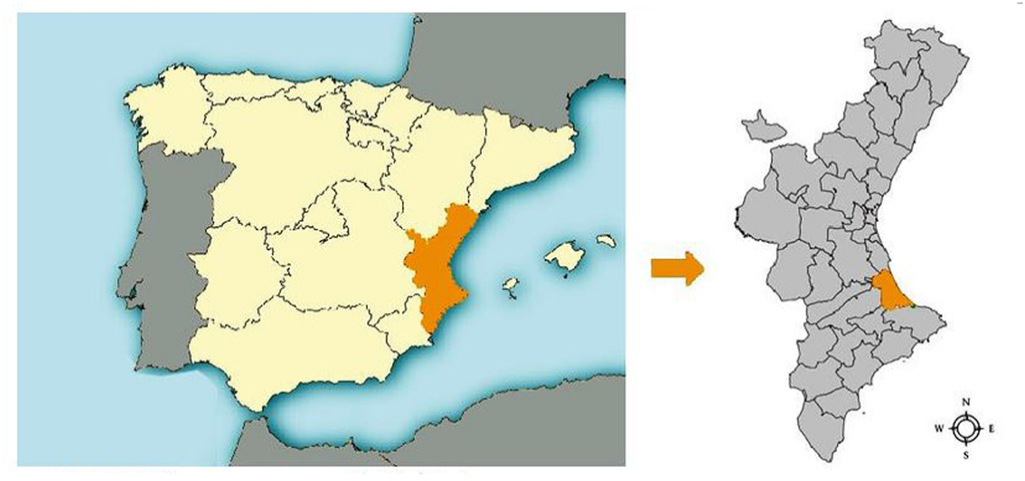
Location of La Safor within Spain.

The first article from Roman-Sanchez et al. [8] reveals a prevalence of 0.9% considering all cases of *S. stercoralis* infection among all patients admitted to a reference hospital in Gandía (capital of the region of La Safor). Most of the infected patients were elderly male farmers who admitted to having worked barefoot in rice fields.

The second article by Roman-Sanchez et al. [9] provides specific infection rates of *S. stercoralis* among farmers in La Safor, who were specifically targeted for screening because this collective had been previously identified as a risk group. Therefore, a prevalence rate of 12.4% was found. Although this is a community-based study, it was not performed in a general population.

With the aim to assess the geographical distribution and characteristics of *S. stercoralis* infection in this region, a more recent study has been published. A prevalence rate of 0.9% was found in only one municipality (Oliva), and the figures were even lower in other studied municipalities. Most patients were elderly males who had worked in rice fields. As barely no cases among youngsters were found and agricultural practices have changed, the authors concluded that there are no data to support current infestation [6].

Other studies on *S. stercoralis* infection have been published outside of the La Safor region, mainly in big cities (such as Barcelona and Madrid) receiving internal migration. One prospective observational study performed in Catalonia described only two autochthonous cases of *S. stercoralis* infection during the last decade. However, it is not clear whether the diagnosis was only made by serology or by direct parasitological methods [10]. Furthermore, another prospective observational study conducted in Barcelona and published early this year was unable to find a sole autochthonous case [11]. In another study conducted in patients with chronic eosinophilia in Madrid, one possible autochthonous case of *S. stercoralis* infection was found by means of a positive serology [12]. Diagnosis of strongyloidiasis based on serology alone is not very accurate, for it can cross react with other helminth infections (such as *Toxocara* spp.), giving false positive results. Other articles describing autochthonous strongyloidiasis in Spain are case reports, published because of their exceptional nature [4, 5].

In conclusion, the reviews providing data on autochthonous cases of *S. stercoralis* infection in Spain have extracted their evidence from studies performed in a very particular area and highlighted data from a single study among a selected population, which may lead to overestimation of the figures. Spain should be considered to be a nonendemic country for strongyloidiasis because autochthonous cases are anecdotic or restricted to a focal area among elderly male farmers who had worked in rice fields in the past.

## References

[1] Bethony J, Brooker S, Albonico M, Geiger SM, Loukas A, et al (2006) Soil-transmitted helminth infections: ascariasis, trichuriasis, and hookworm. Lancet 367: 1521–1532. 16679166 10.1016/S0140-6736(06)68653-4

[2] Schar F, Trostdorf U, Giardina F, Khieu V, Muth S, et al (2013) Strongyloides stercoralis: Global Distribution and Risk Factors. PLoS Negl Trop Dis 7: e2288 10.1371/journal.pntd.0002288 23875033 PMC3708837

[3] Glinz D, Silue KD, Knopp S, Lohourignon LK, Yao KP, et al (2010) Comparing diagnostic accuracy of Kato-Katz, Koga agar plate, ether-concentration, and FLOTAC for Schistosoma mansoni and soil-transmitted helminths. PLoS Negl Trop Dis 4: e754 10.1371/journal.pntd.0000754 20651931 PMC2907416

[4] Mayayo E, Gomez-Aracil V, Azua-Blanco J, Azua-Romeo J, Capilla J, et al (2005) Strongyloides stercolaris infection mimicking a malignant tumour in a non-immunocompromised patient. Diagnosis by bronchoalveolar cytology. J Clin Pathol 58: 420–422. 15790710 10.1136/jcp.2004.017756 PMC1770632

[5] Martinez-Vazquez C, Gonzalez Mediero G, Nunez M, Perez S, Garcia-Fernaandez JM, et al (2003) [Strongyloides stercoralis in the south of Galicia]. An Med Interna 20: 477–479. 14755904

[6] Alcaraz CO, Adell RI, Sánchez PS, Blasco MJV, Sánchez OA, et al (2004) Characteristics and geographical profile of strongyloidiasis in healthcare area 11 of the Valencian community (Spain). Journal of Infection 49: 152–158. 15236923 10.1016/j.jinf.2004.01.016

[7] Puthiyakunnon S, Boddu S, Li Y, Zhou X, Wang C, et al (2014) Strongyloidiasis-An Insight into Its Global Prevalence and Management. PLoS neglected tropical diseases 8: e3018 10.1371/journal.pntd.0003018 25121962 PMC4133206

[8] Sánchez PR, Guzman AP, Guillen SM, Adell RI, Estruch AM, et al (2001) Endemic strongyloidiasis on the Spanish Mediterranean coast. QJM 94: 357–363. 11435631 10.1093/qjmed/94.7.357

[9] Román-Sánchez P, Pastor-Guzmán A, Moreno-Guillén S, Igual-Adell R, Er-Generoso Ss, et al (2003) High prevalence of Strongyloides stercoralis among farm workers on the Mediterranean coast of Spain: Analysis of the predictive factors of infection in developed countries. Am J Trop Med Hyg 69: 336–340. 14628954

[10] Valerio L, Roure S, Fernández-Rivas G, Basile L, Martínez-Cuevas O, et al (2013) Strongyloides stercoralis, the hidden worm. Epidemiological and clinical characteristics of 70 cases diagnosed in the North Metropolitan Area of Barcelona, Spain, 2003–2012. Trans R Soc Trop Med Hyg 107: 465–470. 10.1093/trstmh/trt053 23783760

[11] Salvador F, Sulleiro E, Sanchez-Montalva A, Saugar JM, Rodriguez E, et al (2014) Usefulness of Strongyloides stercoralis serology in the management of patients with eosinophilia. Am J Trop Med Hyg 90: 830–834. 10.4269/ajtmh.13-0678 24615124 PMC4015573

[12] Fernandez Rodriguez C, Enriquez-Matas A, Sanchez Millan ML, Mielgo Ballesteros R, Jukic Beteta KD, et al (2012) Strongyloides stercoralis infection: a series of cases diagnosed in an allergy department in Spain. J Investig Allergol Clin Immunol 22: 455–457. 23101199

